# Modelling the Effects of Population Structure on Childhood Disease: The Case of Varicella

**DOI:** 10.1371/journal.pcbi.1002105

**Published:** 2011-07-21

**Authors:** Romain Silhol, Pierre-Yves Boëlle

**Affiliations:** 1 Université Pierre et Marie Curie-Paris 6, Paris, France; 2 INSERM, Paris, France; 3 Assistance Publique - Hôpitaux de Paris, Hôpital Saint Antoine, Paris, France; Imperial College London, United Kingdom

## Abstract

Realistic, individual-based models based on detailed census data are increasingly used to study disease transmission. Whether the rich structure of such models improves predictions is debated. This is studied here for the spread of varicella, a childhood disease, in a realistic population of children where infection occurs in the household, at school, or in the community at large. A methodology is first presented for simulating households with births and aging. Transmission probabilities were fitted for schools and community, which reproduced the overall cumulative incidence of varicella over the age range of 0–11 years old.

Moreover, the individual-based model structure allowed us to reproduce several observed features of VZV epidemiology which were not included as hypotheses in the model: the age at varicella in first-born children was older than in other children, in accordance with observation; the same was true for children residing in rural areas. Model predicted incidence was comparable to observed incidence over time. These results show that models based on detailed census data on a small scale provide valid small scale prediction. By simulating several scenarios, we evaluate how varicella epidemiology is shaped by policies, such as age at first school enrolment, and school eviction. This supports the use of such models for investigating outcomes of public health measures.

## Author Summary

Individual-based models of disease transmission have increasingly included detailed demographic data to more accurately describe places where population mix and infection occur. These models may help to understand in more detail heterogeneities in transmission and improve public health decisions. Here, the spread of varicella, a childhood disease, is studied in such a model where spatial and population structures are explicitly modelled. The model focuses on children, organized in households, schools and municipalities, in agreement with census data. The detailed structure of the population used in the model allows for reproducing several observed differences in the epidemiology of varicella, for example, variation in age according to birth rank and place of residence. These results support using detailed models with the eventual aim of improving decisions in public health.

## Introduction

Varicella is endemic in most Western countries where vaccination has not been implemented [1], [2]. More than 90% people are infected with varicella-zoster-virus (VZV) before 12 years of age, but the age-specific seroprevalence of VZV shows large variability between countries [3]. For example, the median age at infection ranges between 2 years in The Netherlands and 6 years in Italy [3].

**Figure 1:**
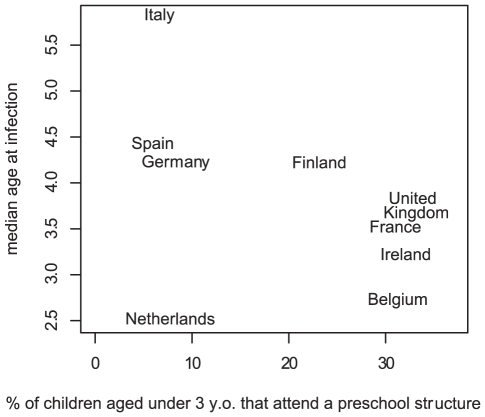
Median age varicella at infection according to pre-school attendance before age 3. Data from OECD and median age at varicella infection in European countries [3], [16].

The explanation of such differences is likely to be found in factors shaping the possibilities of infection in children, such as household structure [4], schools and social behaviours. For example, the median age at varicella infection is smaller in countries where more children attend pre-school (Figure 1). It has also been shown that summer holidays are synchronous with large troughs in varicella incidence in France [5], as children have fewer contacts during these periods. Other characteristics may also shape the epidemiology of the disease, albeit the effect may be more subtle. For example, the age at varicella infection was found to decrease in successively born children of the same household, and an increase in risk followed after school enrolment of the first-born child [6]; the risk of varicella was smaller in less densely populated areas, in agreement with other studies [7]. The effectiveness of interventions, for example vaccination or school exclusion, could be changed by such differences. Using models describing the population and its structure in sufficient detail is the only way to capture this heterogeneity and improve predictions.

Indeed, computational models of disease spread have increasingly tried to more accurately describe places where population mix and infection occur, using detailed demographic data (see for example [8]–[12]). As for now, these models have largely been motivated by studying pandemic influenza or bioterrorist attacks, and none incorporated aging of the population or perpetuation of the disease over several years, because the typical timescale of the epidemics lasted only a few months. Including a realistic demographic process in such models has been described as a challenge[13], and only rarely considered in practice[14].

Indeed, census data typically provide a cross-sectional view of the population, yielding the current distribution of household sizes and age of members. Using such data, it is possible to simulate populations in which household structure and age structure conform with the census (see [15]). However, including births and aging in such a population leads to some difficulties. For example, households where only one child is reported at the time of census contain a mix of single-child households where no other child will be borne, and households in which the younger siblings have not yet been born. To properly account for the demographic process, new births must occur in the latter; but this changes the overall size distribution of households, in turn producing characteristics of the simulated population that may no longer compare to the observed population.

Here, the simulation of a realistic population of children over several years is described, using detailed census data. The spread of varicella is explored in this population, where infection can be transmitted in several locations (household, school and municipality). Model predictions are compared with data formerly obtained from the Corsican children population in 2008 [6], and the impact of some socio-behavioural changes are considered.

## Methods

A spatially explicit, stochastic, agent-based model was developed using detailed demographic data from the island of Corsica. The study population is limited to children with an age of less than 12 years old (y.o.), as varicella is infrequent in older subjects [3], [16], [17]. The model is designed to reflect the main mixing places for children: households, schools, and communities at large.

### Basic components for generating realistic households

**Figure 2:**
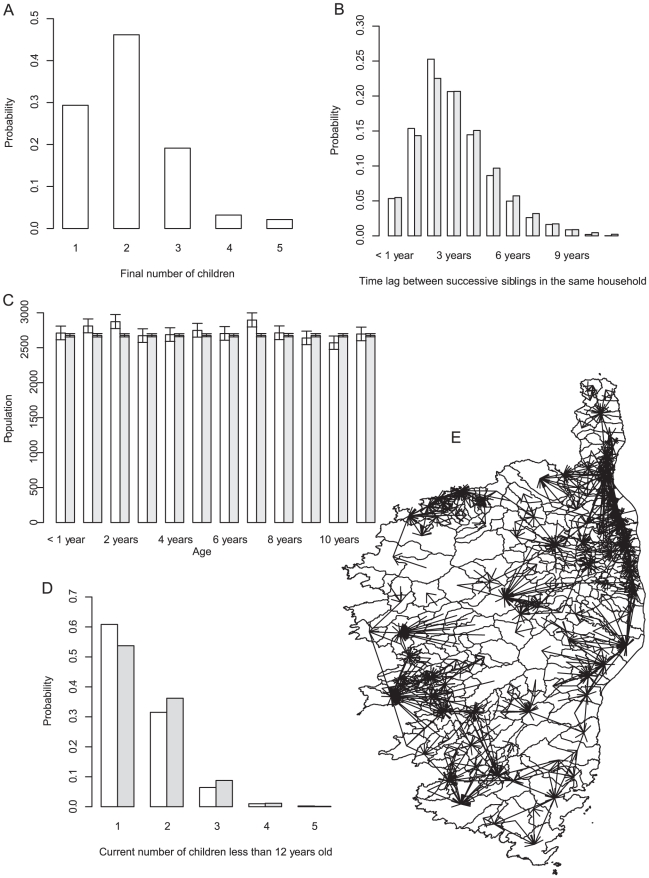
Simulation of a realistic children population in Corsica. (A) Distribution of household final number of children. (B) Observed (white) and simulated (grey) distribution of time lag between successive siblings (in years). (C) Observed (white) and simulated (grey) number of children in Corsica according to age. (D) Observed (white) and simulated (grey) number of children aged less than 12 years old per household. (E) Commuting to school outside the municipality of residence.

Creating households directly from census data, as described above and in [15], does not easily allow maintaining the population structure as aging is introduced into the simulation. For instance, no simple rule allows deciding in which households new children should be borne. For example, Ajelli allocated newborns to new or existing households on the basis of probabilities calculated from current household size and age of children [14], requiring computations at almost each new birth. To circumvent this difficulty, we estimated first the final household size (FHS) distribution from census data, i.e. the number of children in households where no other children will be borne, as well as the age difference (AD) distribution of the time lag between successive siblings in the same household. An estimate of the FHS distribution (see Figure 2A) was obtained by computing the total number of children in households where the oldest child was between 13 and 17 years old, assuming that no additional births were possible in these households. The observed AD was fitted by a gamma distribution (see Figure 2B) and was assumed independent of the birth order within the household.

### Population & school data

The Corsican population (300.000 inhabitants, comprising 35.000 children aged under 12 y.o.) was split over municipalities (n = 360, mean area: 24 km^2^), according to current number of households comprising of at least one children less than 12 years old. Schools (n = 268) were created using data from the French ministry of national education at the corresponding locations. The school capacity (number of children during the school year), and type of school (schools for children with ages from 3 to 7 y.o, for children with ages from 8 to 11 y.o, for all children with ages from 3 to 11 y.o) was also taken into account. Information on commuting and data from the ministry of education were used to allocate children to schools (Figure 2E): The probability of going to school in municipality *j* when residing in municipality *i* was estimated as 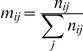
where *n_ij_* were the observed commuting flows for children. Allocation to a school in the municipality was proportional to the expected size of each school, and according to type of school.

### Demographic simulation

The unit of simulation time was the day.

#### Initialisation

The size of a household was sampled in the HFS distribution, and the age of the first born child *a*_1_ was chosen at random. The age *a*_i_ of the *i* ^th^ children in the household was calculated as *a*_i_ = *a*_i−1_ − *d*, with *d* sampled in the AD distribution. With this approach, some children may have a negative age at the first day of the simulation and do not participate in the simulation until their age is larger than 0. This step was repeated until the desired number of households with at least one child less than 12 years old was obtained in each municipality.

#### Aging, births & renewal

At each time step, the age of household members was updated. Children whose age became positive entered the simulation. New households were created (as above) at a rate inversely proportional to the average sojourn time of households in the simulation, and introduced in each municipality according to municipality size so that the number of households was approximately constant over time. The age of the oldest children in newly created households was set to 0.

#### Schooling

Once a year during simulation time (in September), all children who had their 3^rd^ birthday were allocated to a school in a municipality according to the commuting probabilities and school capacity as reported by the ministry of education. Older children changed schools as required according to age. The school calendar, defining days without school (weekends, small holidays and summer holidays) was recreated as in the year 2007.

### Epidemic simulation

The natural history of varicella was described by an M/S/E/I/R compartmental model [18]–[21]. All children were born susceptible to varicella infection. After birth, they entered compartment M with decreasing protection from infection until 6 months of age [22], then compartment S where they were susceptible to infection. In case of infection, the child was first latent (stage E, infected not infectious), then infectious and asymptomatic (stage Ia) followed by infectious and symptomatic when the skin rash finally appears (stage Is). Eventually, all children recovered (stage R). In stage M, protection by maternal antibodies was 95% until month 1, then 75% until week 9, 50% until week 14, 25% until week 20, and 5% until month 6 [22]. The duration of the latent period (stage E) was sampled in a discretized normal distribution with mean = 14 days and standard deviation 2.5 days [23], the infectious asymptomatic period in a discretized gamma distribution with mean = 1.7 days and standard deviation 0.2 days [1], [2] and the subsequent symptomatic period in a discretized normal distribution with mean = 5 days and standard deviation 1 day [1], [2].

#### Disease transmission

The daily probability of infection *P* was calculated according to exposure in the household, the school and the community at large as in [10]: 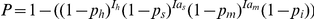 where 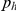, 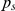,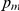 were the daily probabilities of infection by an infectious sibling, an infectious schoolmate, or a child in the municipality. We also included an external probability of infection (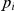) assumed to be small [24]. In the equation above, 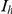 was the number of infectious siblings in the household, *Ia_s_* was the number of infectious but asymptomatic children in a random sample of contacts in the school, and *Ia_m_* was the number of asymptomatic infectious in a random sample of contacts in the municipality. The number of daily contacts in the school was set to the average school class size (25 children, or less in smaller schools) [25], and to 5 in the municipality. Contacts in the municipality represent local contacts which are neither household nor school based. As soon as children were symptomatic, they were removed from the school and the community until recovery, but could still infect their siblings. All simulations started with infection of 5 random individuals. The model was run for 100 years, discarding the 20 first years to avoid transient effects.

### Summary epidemiological outputs from the model

From model simulations, we calculated the following quantities (averaged over 80 successive years):

- Cumulated incidence of varicella according to age (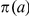, 0<a<12). The percentage of children at each age who have had varicella was determined each September (simulation time). The cumulated incidence of varicella in first-born children and in others, and in rural and urban settings were also calculated: we used census data to define “urban children” and “rural children” as children living in a municipality from the 1^st^ quartile and 4^th^ quartile of municipality population respectively [6].
- Weekly incidence of varicella.
- Place where infection occurred. Each time a child was infected, the source of infection was calculated proportionally to the terms in *P*. For example, at the end of the day, the probability that a new case was infected in his household was:
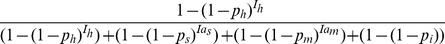

### Estimating parameter values

The parameters 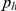,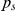,
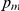
and 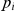
required to run a simulation are not precisely known for varicella. The household secondary attack rate (HSAR), which approximates the pairwise probability of transmission in the household, is approximately 70% for varicella [6], [26]. As this is little changed by household size (69% in households with 2 children, 76% in households with 3 children [6]), we assumed constant pairwise transmission irrespective of household size. Solving the theoretical pairwise transmission probability in the household for this value (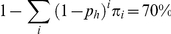 where *π_i_* is the probability distribution of the infectious period duration distribution) led to *p_h_* = 20%.

The other terms were determined using maximum likelihood. More precisely, we computed the likelihood of the model based on the cumulative incidence according to age as 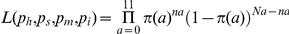, where 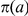 is the model predicted cumulated incidence by age *a*, and *n_a_* the number of children who have had varicella before age *a* out of a total of *N_a_* in a sample of Corsican children [6].

Exploration of the likelihood was done using Latin hypercube sampling [27]. Parameter *p_h_* was fixed at 0.2, *p_i_* was sampled in the range 0–10^−4^ and the two other were sampled between 0 and 1. The Akaike Information Criterion was used to compare models [28].

### Comparison to realistic age-structured (RAS) model

A realistic age-structured model has been implemented as described in [29]. In this simpler model, the Corsican youth population was divided in 14 age groups (0–0.5 years, 0.5–1 year, 1–2 years, 2–3 years,… 11–12 years, 12 years and above). The 0–0.5 year olds were assumed to be immune to infection. We used the POLYMOD data (from the UK) for the contact matrix. Finally, the model was fit to the Corsican cumulated incidence data by maximum likelihood[29].

## Results

The population structure created in the simulation was stable over time (number of households, number of children), and the age distribution of children and current household size matched that of the census data (Figure 2C and 2D).

**Figure 3:**
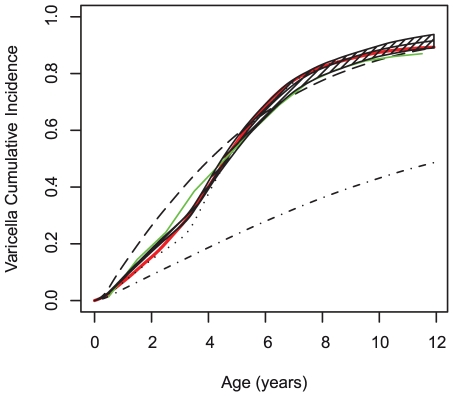
Observed and simulated cumulated incidence of varicella. Observed cumulated incidence of varicella (red), simulated by the RAS model (green), and simulated allowing Households only (dashed dotted dark), Households and Municipalities (dashed dark), Households and Schools (dotted dark), Households and Schools and municipality (plain dark). Hatches correspond to the 95% CI of the “Households and Schools and Municipality” model.

We explored four versions of the model, allowing different locations for mixing in each version. Each time, we selected the parameter set leading to the best fit in terms of likelihood (Figure 3 and Table 1). Using this set of parameters, we predicted the cumulated incidence in first-born and other children, as well as in rural and urban areas. The corresponding curves were compared with those obtained in the actual population.

**Table 1:** Model parameters values and goodness of fit based on cumulated incidence of varicella.

Mixing levels allowed in the model	Homogeneous iage-structured mixing	Household; External	Household; Municipality; External	Household; School; External	Household; School; iMunicipality; External
(Model name)	(RAS)	(HE)	(HME)	(HSE)	(HSME)
*p_h_ (fixed)*		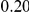	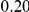	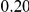	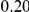
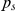		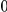	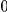	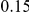	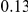
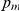		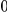	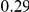	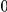	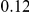
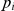		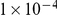	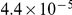	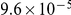	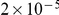
Log-Likelihood	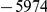	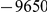	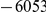	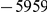	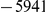
AIC		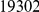	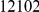	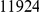	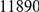
Source of infection					
Household		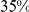	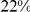	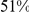	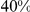
School		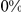	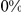	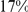	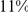
Municipality		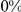	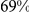	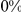	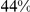
External		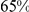	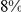	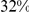	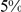

### Cumulative incidence with age and sources of mixing

Using the RAS model, it was possible to simulate cumulative incidence data close of what was observed in Corsica. However, the fit of this model, as measured by the likelihood, was worse than that derived in the best fitting individual-based models (Figure 3 & Table 1).

In the individual-based model, when the probabilities of being infected with varicella in the municipality and in the school were set to 0 (model HE), it was not possible to obtain a good fit with the cumulated incidence profile with age. In the best fitting combination, even when large external transmission was allowed, the cumulated incidence (CI) at age 12 was 48%, short of the 90% observed (Figure 3). In this model, 65% of infections occurred outside households (see Table 1).

We then included transmission in the municipality, but not in schools (model HME). The fit improved, with cumulative incidence of varicella reaching 90% by 12 years of age. The percentage of infections occurring outside households and the municipality was 8% (Table 1). However, in this model, the cumulated incidence increased too quickly with age as compared to the observed data. Indeed, the CI was 32% at 2 years old and 54% at 4 years old, compared with 21% and 48%.

The model with transmission in schools, but not in the municipality (model HSE), provided a better global fit as judged by the AIC. However, this time, the CI increased too slowly in children aged less than 3 years old, reaching 18% compared with 20% in the real data. Moreover, the CI at 12 years old was 92%, more than the observed 89%.

Finally, the model allowing transmission in both schools and municipalities (model HSME) provided the best fit (i.e. smallest AIC, see Table 1). The simulated cumulated incidence of varicella was almost undistinguishable from the observed data. The range of parameter values leading to small differences in AIC (<2) was narrow, between 0.114 and 0.122 for *p_m_*, between 0.128 and 0.134 for *p_s_* and between 1.9×10^−5^ and 2.1×10^−5^ for *p_i_*. The proportion of infections due to external exposure was reduced at 5.5% (Table 1).

### Comparing detailed model predictions with observed data

**Figure 4:**
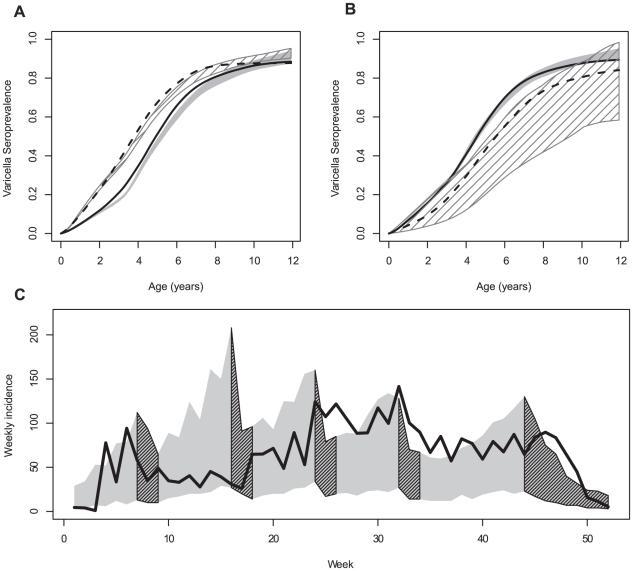
Best fitted model predictions: cumulated incidences and weekly incidence. (A) Cumulated incidence in first-borns (observed: plain, simulated: grey zone) and others (observed: dashed, simulated: hatched zone). (B) Cumulated incidence in urban (observed: plain, simulated: grey zone) and rural municipalities (observed: dashed, simulated: hatched zone). (C) Observed weekly incidence (plain) and simulated (grey zone) starting from the first week of September to the end of August. Hatches correspond to school holidays. In all simulations, the maximum and minimum from over 100 years of simulation are reported.

Using the best fitting model (HSME), the cumulative incidence of varicella was predicted in two special cases: in first-born and in other children; and in rural and urban settings. Figure 4 show that the model predicting cumulative incidence with age matched the characteristics of the observed data. Indeed, the age difference at varicella observed among siblings of the same family was present (Figure 4A), with an increase in incidence after 3 years of age in first-born children; the difference between rural and urban settings was also present with varicella occurring at a later age in children living in more rural settings.

When all levels of mixing were not allowed, the model failed to reproduce these quantities. For example, when the municipality was not included, although the simulated overall cumulated incidence was close to that observed in Corsican children, the cumulated incidence in first-born children was largely underestimated at three years old: 8% at 3 years old (not shown), compared with 22% in the observed data.

The average weekly incidence agreed with those reported by the Sentinelles network system [30]. The simulated incidence time series showed a reduction in incidence that was associated with holidays, with a pronounced trough during the summer period (Figure 4C). However, the observed data was not entirely reproduced, as the model predicting incidence was the highest after summer holidays and decreased during the school year, when the observed data suggested that it was the reverse.

### Exploring the effect of changes in behaviour

**Figure 5:**
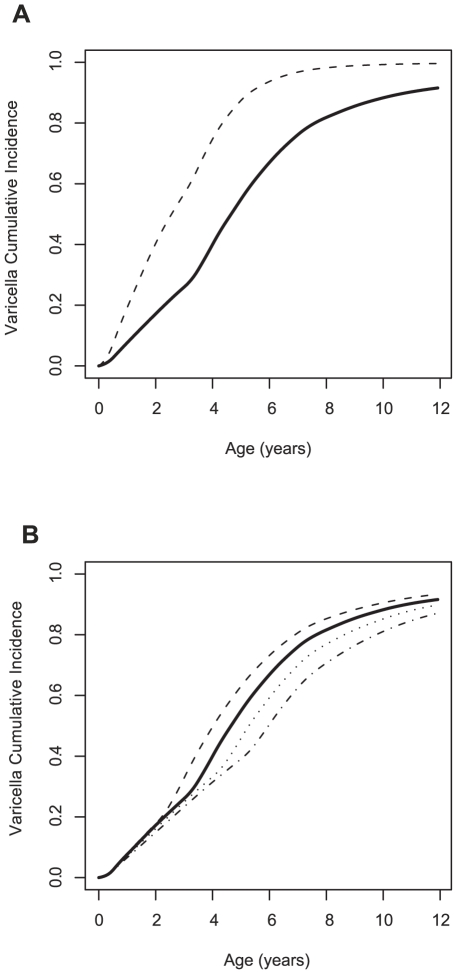
Varicella CI according to different scenarios. (A) Varicella CI according to school exclusion (school exclusion - current: plain, no school exclusion: dashed). (B) Varicella CI according to age at first-school enrolment: at 3 y.o. (current policy) (bold), at 2 y.o. (dashed), at 4 y.o. (dotted), at 5 y.o. (dash dotted).

In France, infected children are excluded from school as soon as varicella symptoms are recognized. We explored how this public health measure impacted the spread of varicella. Simulations were run assuming that infected children remained present in the community and school until they were cured, with the same level of infectivity as during the asymptomatic stage. As a consequence, age at varicella decreased in the whole population, with median age at infection shifting from 4.7 to 2.6 years old (Figure 5A). This would also lead to almost all children being infected with varicella before age 12, compared with approximately 90% in the field data [6]. Next, we explored how age at schooling may explain the variability in varicella age-seroprevalence between countries where vaccination is not implemented. We simulated scenarios in which age at first school enrolment was set at 2, 4, 5 and 6 years old. The effect of this change is presented in Figure 5B. The median age at infection increased by about 8 months for each one-year increase in age at school enrolment.

## Discussion

In this paper, we have shown that including detailed population structure in models of varicella transmission allowed us to reproduce the cumulated incidence of varicella according to age. A better fit was obtained than with a realistic age structured model, even using mixing matrices based on real contacts. Importantly, specific features observed in varicella epidemiology were reproduced owing to the detailed structure of the model. This included, for example, differences in age at varicella according to birth rank and place of residence, indicating that the rich structure built into epidemiologic models using census data leads to improved models regarding disease spread.

Modelling varicella, or other childhood diseases, requires simulating populations over several years. Indeed, seroprevalence studies show that most infections occur during the first 12 years of life [3], [16], [17]. It is therefore required to simulate realistic children populations over several years. Here we showed that it could be achieved by knowing only the number of households in each municipality, the household final size distribution and the age difference distribution between successive siblings. Throughout the entire simulation, the simulated population agreed with census data regarding age of children, household sizes, and movement flows (see Figure 2). To determine the household final size distribution, we focused on households where the oldest child was between 13 and 17. A sensitivity analysis indicated that the bottom threshold could be chosen in the range of 10–16 with little change in the estimated HFS distribution. The average final size of the household was 1.9, in agreement with the total fertility rate in women who have had at least one child [31]. Other demographic processes, such as change in household structure due to divorce or remarriage, were not modelled; neither were the opening or closing of schools, which happens depending on the number of children in small municipalities.

Varicella provided an excellent case study, since it is a common childhood disease in France (as universal vaccination is not recommended) and surveillance data is available, however the model could however easily be applied to other childhood infectious diseases. The varicella natural history description was standard [18], with an additional split of the infectious period according to the presence of symptoms. This allowed to model the prodromic infectious phase, often reported for varicella, where infectivity increases in the few days before a rash is present [1], [2]. As seen in the simulations, these 1 or 2 days were important for transmission: indeed, the models not including school and municipality transmission failed to reproduce the incidence data. Truly asymptomatic varicella was not modelled as it is rare [32] and presumably less infectious [33].

One issue in the present modelling was how to initialise the population regarding susceptibility to the disease. Indeed, the susceptibility of siblings or schoolmates is not independent, since the disease is transmissible. We used two approaches: (1) start with an entirely susceptible population or (2) randomly assign a susceptibility status according to the observed cumulative incidence with age [6]. In either case, after discarding the initial first 20 years of simulation the results were not sensitive to the actual method of initialisation, although the simulated cumulated incidence rate with age was more quickly stabilized with the second method. A second issue was to quantify and average over stochastic variation. Averaging over at least 80 years of simulation was required in this respect.

The choice of Corsica to build the model was motivated by the availability of epidemiological data for comparison, nevertheless changing the input census data would make it possible to use the model in other settings. A child contact network was described as household, school and municipality. The detailed description of these places structured the possibilities of interaction according to age and space, in place of mixing matrices used in other models [29], [34], [35]. In Table 2, we report how changing the number of contacts at school or in the municipality changed our results. There was altogether little effect on the overall fit. Unexpectedly, the proportion of varicella cases due to school contacts decreased with increasing contacts at school. However, more contacts in school led to increased introduction of cases in households and the municipalities (spatial diffusion), where transmission could start, and lead to more cases. The school size (median school size = 63 children) further limited its overall influence. Other typical mixing places for young children may include day care, but this is almost inexistent in Corsica (less than 200 children overall) and was not considered here. How transmission depends on household size is a current matter of debate, and our model assumed a constant pairwise transmission in household, as suggested by [6]. We found that this hypothesis was in good agreement with the data: In households comprising 2 susceptible children before the introduction of the virus, the observed distribution of the number of cases was 1 case: 31%, 2 cases: 69%, the simulated distribution was 1 case: 27%, 2 cases: 73% (p.value = 0.93); whereas in households comprising 3 susceptible children before the introduction of the virus, the observed distribution of the number of cases was 1 case: 12%, 2 cases: 23%, 3 cases: 65%, the simulated distribution was 1 case: 8%, 2 cases: 24%, 2 cases: 68% (p.value = 0.99).

**Table 2:** Model sensitivity analysis on the number of contacts in the municipality and in the school.

Number of contacts	Log-likelihood of the best-fit model	% of cases occurring in households	% of cases occurring in school	% of cases occurring in municipalities	% of cases occurring outside
3 contacts in the municipality	−5941.84	40	11	44	5
10 contacts in the municipality	−5941.12	40	11	45	4
20 contacts in the municipality	−5941.05	40	11	45	4
50 contacts in the municipality	−5941.55	40	11	45	4
100 contacts in the municipality	−5941.71	40	11	46	3
10 contacts at school	−5941.52	39	14	43	4
15 contacts at school	−5941.34	40	12	45	3
30 contacts at school	−5941.12	40	11	46	3
40 contacts at school	−5941.66	42	10	44	4
75 contacts at school	−5941.58	42	8	46	4

The basic reproduction number, corresponding to the number of secondary cases caused by one case in a totally susceptible population, (R_0_) was approximately 4 (average over 500 simulations with 1 initial random case). This value was in the low end among European countries [3]. There was large variation depending on whether the initial children attended school (4.4 secondary cases) or not (1.3 secondary case). Using the RAS model, this estimate was close to 14. This shows that the reproduction number is indeed very dependent on how contact are calculated, as previously noted by other authors for varicella [29], [36]. As seen in Figure 2C, the school commuting network is essentially based on geography, most children going to school in their own or a neighbouring municipality. The difference between contacts at school and in the municipality is therefore mostly due to the age distribution of contacts. Even if the main sources of mixing were included, it was necessary to include an external force of infection. When this was not allowed, the disease rapidly went extinct especially during the summer holidays. The persistence of a disease requires sufficient a community size, as is well-known in the case of measles [37], which may not be met in Corsica. Introducing an external source of infection (*p_i_*) was necessary to perpetuate varicella. Indeed, persons previously infected with the varicella virus may start shedding the virus again, for example during zona episodes, and originate transmission chains. Indeed, Zona incidence is approximately constant over time in Corsica (see http://www.sentiweb.org), and together with case importation, this supports introducing an external force of infection for varicella in small populations [38].

The model required estimating only 3 parameters corresponding to daily transmission probabilities, and provided a very good fit to the data. In the best fitting case, the daily probability of pairwise transmission in the household was approximately 17%, and it was 13% in the school and 12% in the municipality. Interestingly, these probabilities are consistent with the rate of varicella transmission (0.00133/minute) derived from models based on time use data [34]. Indeed, a daily probability of 17% is obtained for a duration of contact of 140 minutes (calculated as 
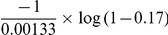
), when the average daily cumulated time of contact between 2 children aged less than 10 years old is indeed between 100 and 200 minutes [34]. The model did not reproduce all features of observed incidence with time. Overall, the observed incidence of varicella showed an increasing trend during the school year, with the highest point being before the summer holidays (see Figure 4C), but the model predicted incidence, while highly variable, that often showed the opposite trend. To properly reproduce this feature, transmission in schools, in municipalities or the “external transmission” should be lower in winter and larger in the spring: Such seasonality could be the result of impaired transmission in cold weather [39], or of fluctuations in the incidence of zona [40].

In conclusion, detailed simulation of realistic children populations over several years may improve the study of childhood disease transmission. Further comparisons with compartmental models using realistic mixing matrices are necessary to identify the best approaches to help public health decisions.
